# Effects of Tryptophan Supplementation and Exercise on the Fate of Kynurenine Metabolites in Mice and Humans

**DOI:** 10.3390/metabo11080508

**Published:** 2021-08-03

**Authors:** Paula Valente-Silva, Igor Cervenka, Duarte M. S. Ferreira, Jorge C. Correia, Sebastian Edman, Oscar Horwath, Benjamin Heng, Sharron Chow, Kelly R. Jacobs, Gilles J. Guillemin, Eva Blomstrand, Jorge L. Ruas

**Affiliations:** 1 Department of Physiology and Pharmacology, Biomedicum Karolinska Institutet, 171 77 Stockholm, Sweden; paula.da.silva@ki.se (P.V.-S.); igor.cervenka@ki.se (I.C.); duarte.ferreira@ki.se (D.M.S.F.); jorge.correia@ki.se (J.C.C.); 2 Department of Physiology, Nutrition and Biomechanics, Swedish School of Sport and Health Sciences, 114 33 Stockholm, Sweden; sebastian.edman@gih.se (S.E.); oscar.horwath@gih.se (O.H.); eva.blomstrand@gih.se (E.B.); 3 Neuroinflammation Group, Department of Biomedical Sciences, Faculty of Medicine and Health Sciences, Macquarie University, Sidney, NSW 2109, Australia; benjamin.heng@mq.edu.au (B.H.); sharron.chow@mq.edu.au (S.C.); kelly.r.jacobs@gmail.com (K.R.J.); gilles.guillemin@mq.edu.au (G.J.G.)

**Keywords:** dietary supplements, exercise, tryptophan, kynurenine metabolites, energy metabolism, skeletal muscle, behavior

## Abstract

The kynurenine pathway of tryptophan (TRP) degradation (KP) generates metabolites with effects on metabolism, immunity, and mental health. Endurance exercise training can change KP metabolites by changing the levels of KP enzymes in skeletal muscle. This leads to a metabolite pattern that favors energy expenditure and an anti-inflammatory immune cell profile and reduces neurotoxic metabolites. Here, we aimed to understand if TRP supplementation in untrained vs. trained subjects affects KP metabolite levels and biological effects. Our data show that chronic TRP supplementation in mice increases all KP metabolites in circulation, and that exercise reduces the neurotoxic branch of the pathway. However, in addition to increasing wheel running, we did not observe other effects of TRP supplementation on training adaptations, energy metabolism or behavior in mice. A similar increase in KP metabolites was seen in trained vs. untrained human volunteers that took a TRP drink while performing a bout of aerobic exercise. With this acute TRP administration, TRP and KYN were higher in the trained vs. the untrained group. Considering the many biological effects of the KP, which can lead to beneficial or deleterious effects to health, our data encourage future studies of the crosstalk between TRP supplementation and physical exercise.

## 1. Introduction

Adaptation to endurance exercise training depends on a variety of changes in tissues such as the heart, skeletal muscle, immune system, and the brain (among other). For that reason, the benefits of exercise training to human health range from improved energy metabolism and muscle function to better mental health. Strategies to improve or accelerate exercise adaptations usually change variables related to the type and intensity of training, nutritional choices, or a combination of both. In this context, several nutritional supplements have been and continue to be explored [1]. Among those, the essential amino acid tryptophan (TRP) has always attracted considerable interested, as it is the precursor for the biosynthesis of serotonin and melatonin, and therefore a regulator of mood [2]. In addition, TRP catabolism through the kynurenine (KYN) pathway (KP) generates a variety of metabolites with diverse biological activities and can ultimately lead to de novo NAD^+^ synthesis in some cell types [3]. KP metabolites impact the function of several tissues and organs including brain, immune system, adipose tissue, skeletal muscle, and gastrointestinal tract. Since some of these metabolites have counteracting mechanisms of action their relative levels dictate the biological output of the pathway. Thus, understanding how the KP is regulated is an area of great interest for a growing number of scientific fields such as oncology, immunology, psychiatry, and metabolism (for review see [3]).

The first step of the KP (Supplementary Figure S1A) consists of the biotransformation of TRP into KYN by the enzymes tryptophan 2,3-dioxygenase (TDO) or indoleamine 2,3-dioxygenase (IDO) 1 and 2. KYN is an agonist of the aryl hydrocarbon receptor (AhR), a ligand-regulated transcription factor involved in xenobiotic metabolism, immunity, and cell differentiation [4,5]. Since KYN (and kynurenic acid, KYNA)-mediated activation of AhR results in reduced inflammation and immune surveillance, this pathway has been targeted for the development of cancer immunotherapy drugs. KYN can then be converted to KYNA by kynurenine aminotransferases (KATs), or to 3-hydroxykynurenine (3-HK) by the enzyme kynurenine monooxygenase (KMO). 3-HK can generate xanthurenic acid (XA) or 3-hydroxyanthanilic acid (3-HAA), through the actions of KMO and kynureninase (KYNU), respectively. Another way to generate 3-HAA is by KMO-mediated hydroxylation of anthranilic acid (AA), which in turn originates from KYNU-mediated catabolism of KYN. Finally, through a series of enzymatic and/or spontaneous chemical reactions, 3-HAA can produce picolinic acid (PA) and quinolinic acid (QA), which can be further transformed into NAD^+^ (Supplementary Figure S1A) [6].

In addition, the metabolites 3-HK and QA are strong reactive oxygen species (ROS) generators, while KYNA is a ROS scavenger [7,8,9]. Similarly, QA exerts excitotoxicity through N-methyl-D-aspartate receptor (NMDAR) agonism, whereas KYNA is an antagonist for the same receptor [10,11]. There are several factors that change the expression levels of KP enzymes, and therefore the levels of the resulting metabolites. Among the most well-established activators of the KP are pro-inflammatory cytokines and cortisol [12,13].

We and others have previously shown that endurance exercise training in mice and humans can change the KP by inducing the expression of skeletal muscle KATs, which convert accumulating KYN into KYNA, increasing muscle energy efficiency [14,15,16] and protecting the brain from stress-induced neurotoxic insult [14]. In addition, KAT expression has also been shown to increase in immune cells with acute exercise [17]. The resulting increase in circulating KYNA levels has the additional effects of increasing adipose tissue energy expenditure and promoting an anti-inflammatory immune cell profile [18]. However, exercise-mediated regulation of the KP is dependent on the activation of skeletal muscle peroxisome proliferator-activated receptor gamma coactivator-1alpha1 (PGC-1α1), an exercise-induced transcriptional coactivator that in turn enhances KAT gene transcription in the myofibers. For that reason, we hypothesized that an excess of TRP intake in untrained individuals, could result in increased levels of toxic KP metabolites. In this study, we aimed to understand how TRP supplementation affects the KP in mice with or without access to running wheels, and how that affects their metabolism and behavior.

## 2. Results

**Figure 1:**
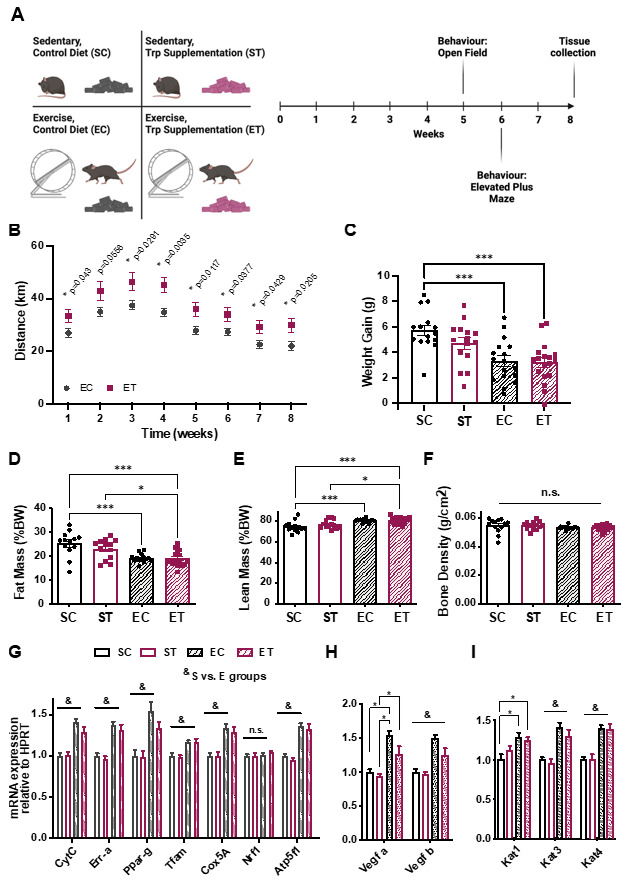
A tryptophan-supplemented diet increases physical activity in mice. (**A**) Schematic representation of the experimental design for the 8-week TRP-supplemented diet and exercise intervention. C57bl/6J mice were divided in 4 groups: sedentary with control diet (SC, 0.2% TRP; *n* = 13), sedentary with diet enriched in TRP (ST, 1% TRP; *n* = 13), with access to a running wheel and control diet (EC, 0.2% TRP; *n* = 15), with access to a wheel and TRP-enriched diet (ET, 1% TRP; *n* = 17). Behavior tests were performed at weeks 5 (open field) and 6 (elevated plus maze). Tissue collection was performed at week 8. (**B**) Distance ran in the wheels during the 8-weeks. (**C**) Weight-gain after the conclusion of the experiment. DEXA-scanning determination of (**D**) fat mass, (**E**) lean mass, and (**F**) bone density at the end of 8 weeks. Gastrocnemius muscle was used for qRT-PCR analysis of gene expression related to energy metabolism (**G**), angiogenesis (**H**), and kynurenine to kynurenic acid conversion enzymes (**I**). Gene expression data is normalized to the expression of a housekeeping gene (hypoxanthine phosphoribosyltransferase, HPRT) and presented as relative to sedentary control (SC). When not indicated *n* = 12–17, due to outlier exclusion. Data is presented as mean values ± standard error of the mean (SEM). * *p* < 0.05; *** *p* < 0.001; & Sedentary vs. Exercised *p* < 0.05.

To evaluate the effects of a diet with TRP supplementation on mice with or without access to in-cage freewheel running, we started by designing two isocaloric diets with defined amounts of all components (Supplementary Figure S2). A control diet containing 0.2% TRP (*w/w*), and a TRP-supplementation diet with five-times higher levels of this amino acid. C57bl/6J mice were then divided into four groups: Control diet without wheel (sedentary, control diet–SC), control diet with wheel (exercise, control diet–EC), sedentary, with TRP supplementation (ST), and TRP supplementation with access to wheel (ET) (Figure 1A). Mice were then closely monitored for eight weeks (Figure 1A) by using a variety of molecular and functional analyses. From the beginning of the experiment, it was clear that ET mice ran more than the EC group (Figure 1B), in terms of both the duration of activity and intensity of wheel running (Supplementary Figure S1B).

However, this difference in running activity was not sufficient to yield differences in weight-gain (Figure 1C), fat mass (Figure 1D), or lean mass (Figure 1E). As expected, the groups given access to a wheel gained less weight and showed reduced adiposity at the end of the eight-week period, regardless of diet (Figure 1C–E). Bone density was unchanged in all groups (Figure 1F).

**Figure 2:**
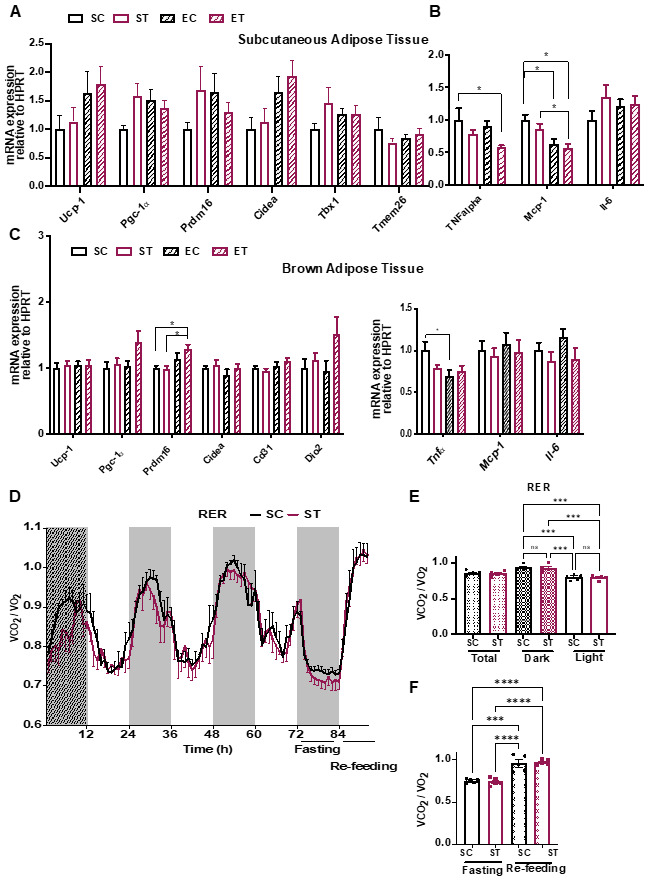
Metabolic analysis of molecular and phenotypic changes in energy expenditure. (**A**) qRT-PCR analysis of the expression of genes related to energy expenditure in subcutaneous adipose tissue from the different experimental groups, as described in Figure 1A (*n* = 11–17). (**B**) qRT-PCR analysis of genes related to inflammation in subcutaneous adipose tissue (*n* = 11–17). (**C**) qRT-PCR analysis of gene expression in brown adipose tissue. Gene expression data is normalized to the expression of a housekeeping gene (hypoxanthine phosphoribosyltransferase, HPRT) and presented as relative to sedentary control (SC) (*n* = 11–17). (**D**) Mice (*n* = 5 per group) were fed in their home cages for 5 weeks with either control diet (SC, 0.2% TRP) or TRP enriched diet (ST, 1% TRP). At week 6 of diet, mice were individually housed in the TSE PhenoMaster home cage system for 5 days with ad libitum access to food and water except for a 12 h fasting period during the last dark cycle (Fasting), followed by 8 h re-feeding (Re-feeding). Respiratory Exchange Ratio (RER) is calculated as the ration between the amount of produced CO_2_ (VCO_2_) and consumed oxygen (VO_2_). Quantification of the data shown in D for the (**E**) Dark and Light cycles or the (**F**) Fasting and Re-feeding period. Data is presented as mean values ± standard error of the mean (SEM). * *p* < 0.05; *** *p* < 0.001; **** *p* < 0.0001.

Since we observed a difference in duration and intensity of wheel running between the EC and ET groups (Figure 1B and Supplementary Figure S1B), we next performed targeted gene expression analysis by qRT-PCR in the gastrocnemius muscles of all groups, to evaluate if the TRP supplementation influenced markers of muscle adaptation to aerobic exercise. As expected, mice with access to a wheel showed increased expression levels of genes related to mitochondrial function and oxidative metabolism (Figure 1G) and angiogenesis (Figure 1H). Again, diet had no statistically significant influence in these changes in gene expression. Since KAT expression was moderately increased by exercise (Figure 1I), and physical exercise is known to induce non-shivering thermogenesis and reduce inflammation in adipose tissue, we analyzed subcutaneous (epididymal) white adipose tissue (WAT) and interscapular brown adipose tissue (BAT) for molecular signs of “browning” and inflammation [19]. In WAT, we observed a trend toward higher levels of several genes involved in thermogenesis (Figure 2A) and a significant decrease in the expression of pro-inflammatory markers such as tumor necrosis factor alpha (Tnf*α*) and monocyte chemotactic protein 1 (Mcp1; Figure 2B). BAT showed higher expression levels of the browning-associated gene PR domain-containing protein 16 (Prdm16), and a reduction in Tnf*α* (Figure 2C). Together, these results indicate that the different diets per se did not influence skeletal muscle or adipose tissue metabolism, but that exercise elicited some of the expected adaptations in both types of tissues.

To complement these molecular analyses, we performed a parallel experiment in which C57bl/6 mice were fed the control (SC) or the TRP supplementation (ST) diet for six weeks. During the last week, mice were placed in metabolic cages for continuous monitoring of O_2_ consumption, CO_2_ production, and food and water intake (Figure 2D–F and Supplementary Figure S3). After a 12 h acclimation stage, parameters were recorded during 60 h, ending with a 12 h fasting and an 8 h re-feeding period (to assess metabolic flexibility). Other than the expected differences between the dark and light cycles, no differences were observed between the control or the TRP supplementation diet groups. Of note, and as opposed to the effects we observed on wheel running (Figure 1B) locomotion (i.e., general in-cage activity) was not affected by the TRP diet (Supplementary Figure S3C). In line with this, no differences were observed in intra-peritoneal glucose tolerance tests (Supplementary Figure S3D). In conclusion, the TRP supplementation diet (at the levels used) did not affect systemic energy metabolism in C57bl/6 mice.

In addition to evaluating the potential metabolic effects of TRP supplementation, we aimed to understand how the different diet and exercise combinations affected the fate of TRP-KYN metabolites. Taking in consideration the relatively short circulation half-lives of KP metabolites [18,20], we started by determining the optimal time to terminate the experiment, considering the beginning of the light phase in our animal facility. Interestingly, we determined that the plasma levels of KP metabolites decrease rapidly as soon as the mice reduce their food intake (Supplementary Figure S4). Indeed, whereas we could observe a significant increase in the levels of all the quantified KP metabolites at the beginning of the light cycle (in this case 6 AM), that could no longer be seen only 4 h later (i.e., 10 AM). These results really highlight the importance of considering time of sample harvest vs. the intervention in question, when it comes to KP metabolite determination.

Taking into consideration the data shown in Supplementary Figure S4, in order to process all the animals in the different experimental groups, mice on the eight-week TRP supplementation diet with or without access to running wheels, were euthanized between 6 and 8 AM.

**Figure 3:**
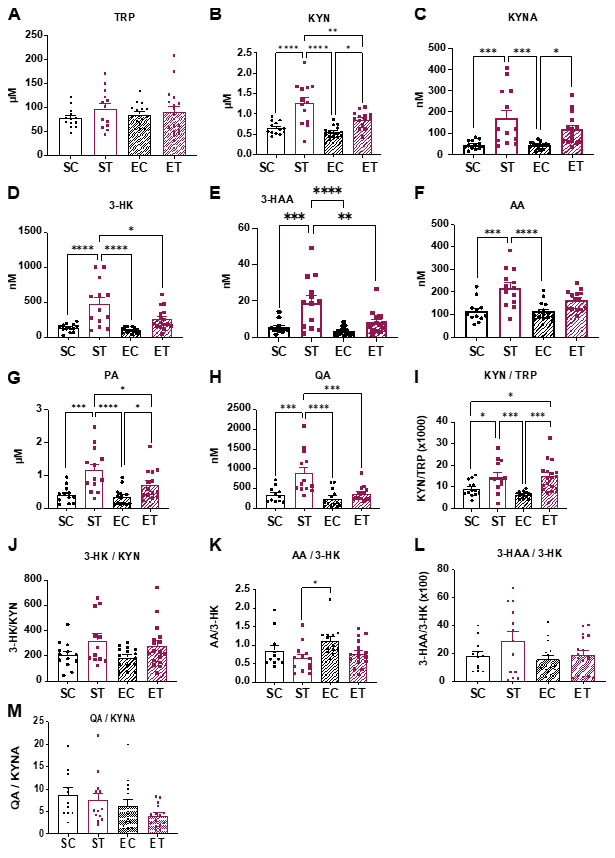
Free wheel running reduces the circulating levels of neurotoxic kynurenine metabolites. Following 8 weeks of control or TRP-enriched diet, without or with access to free wheel running (as described in Figure 1A; *n* = 11–17), mice were sacrificed within 2 h of the start of the animal facility light cycle. Blood was collected and plasma was used for quantification of the indicated metabolites. (**A**) TRP, Tryptophan. (**B**) KYN, Kynurenine. (**C**) KYNA, Kynurenic acid. (**D**) 3-HK, 3-Hydroxykynurenine. (**E**) 3-HAA, 3-Hydroxyanthranilic acid. (**F**) AA, Anthranilic acid. (**G**) PA, Picolinic acid. (**H**) QA, Quinolinic acid. (**I**–**M**) Ratios between the concentrations of the indicated metabolites. *Y* axis shows multiplication factors when used. Data is presented as mean values ± standard error of the mean (SEM). * *p* < 0.05; ** *p* < 0.01; *** *p* < 0.001; **** *p* < 0.0001.

Plasma was collected and used to determine KP metabolite concentrations (Figure 3). Although we could not detect significant differences in circulating TRP levels, all KP metabolites were elevated in the ST vs. the CT group (Figure 3A–H). Interestingly, when compared to the ST group, the ET group showed lower levels of KYN, 3-HK, 3-HAA, and QA (Figure 3B,D,E,H).

Considering the exercise-induced reduction in the neurotoxic branch of the KP, we next used some widely used tests to detect any anxiety-, mood-, or stress-related behaviors in rodents. Since the animals in the different sedentary, exercise, and diet groups were single caged, and isolation per se is known to elicit depressive-like behaviors, we added another group of sedentary control animals fed either the control (GC) or the TRP-supplementation diet (GT), but group caged.

**Figure 4:**
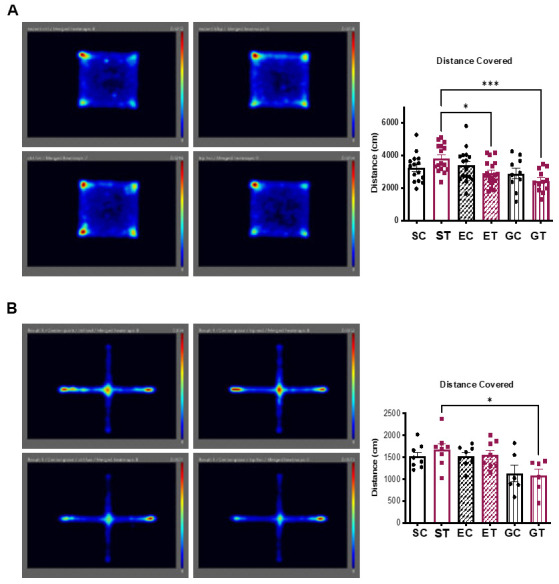
Moderate tryptophan-supplementation does not affect mouse behavior. As outlined in Figure 1A, the different mouse groups were submitted to (**A**) open field and (**B**) elevated plus maze tests. In addition, we added two other sedentary control groups consisting of group-caged animals fed the control (GC) or the TRP-supplementation diet (GT). Pictures show the merged images obtained for the different animals analyzed in the same round of tests (*n* = 6 to 9). Bar graphs show the average quantification of distance covered (*n* = 6 to 17) during the corresponding test. Data is presented as mean values ± standard error of the mean (SEM). * *p*< 0.05; *** *p* < 0.001.

**Figure 5:**
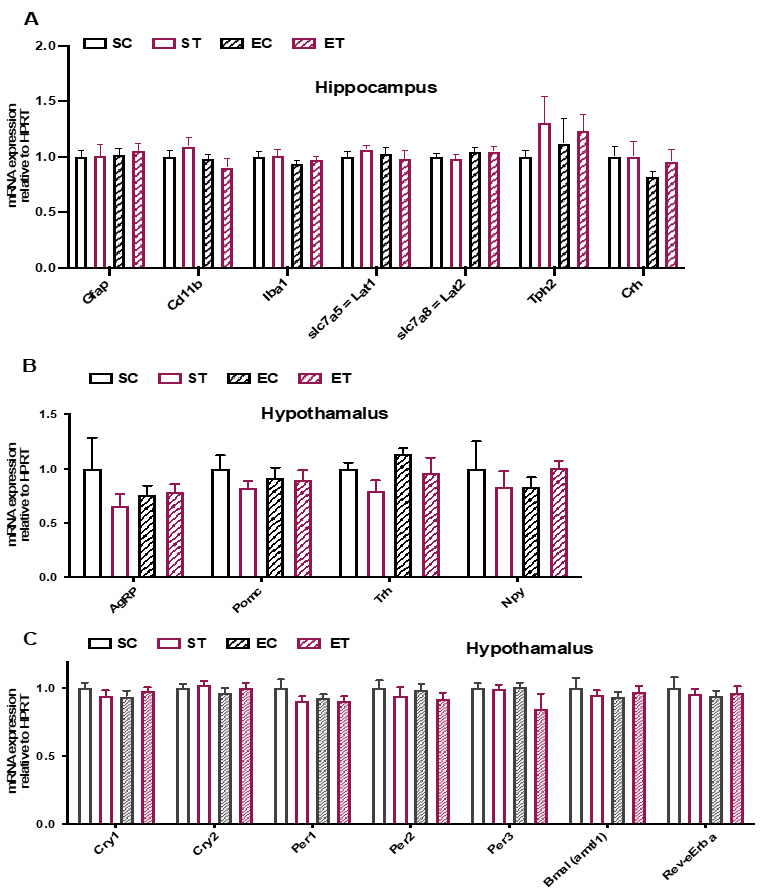
Analysis of hypothalamic and hippocampus gene expression related to changes in stress and food intake. Following 8 weeks of control or TRP-enriched diet, without or with access to free wheel running (as described in Figure 1A), mice were sacrificed within 2 h of the start of the animal facility light cycle. (**A**) qRT-PCR analysis of the expression of genes related to inflammation, tryptophan transport and anxiety in hippocampus. (**B**) qRT-PCR analysis of the expression of genes related to feeding behavior in hypothalamus. (**C**) qRT-PCR analysis of the expression of genes related to circadian regulation in hypothalamus. Gene expression data is normalized to the expression of a housekeeping gene (hypoxanthine phosphoribosyltransferase, HPRT) and presented as relative to sedentary control (SC). Data is presented as mean values ± standard error of the mean (SEM).

Animals were tested in an open field (Figure 4A and Supplementary Figure S5) and in an elevated plus maze test (Figure 4B and Supplementary Figure S6). Overall, we did not observe any significant differences between the groups, indicating that the exercise-mediated reduction in circulating KP metabolites, was not sufficient to affect mouse behavior (at least with the tests used). In line with these results, we did not detect any differences in the expression of hypothalamic or hippocampus genes related to changes in anxiety, inflammation, feeding behavior, and circadian regulation (Figure 5).

**Figure 6:**
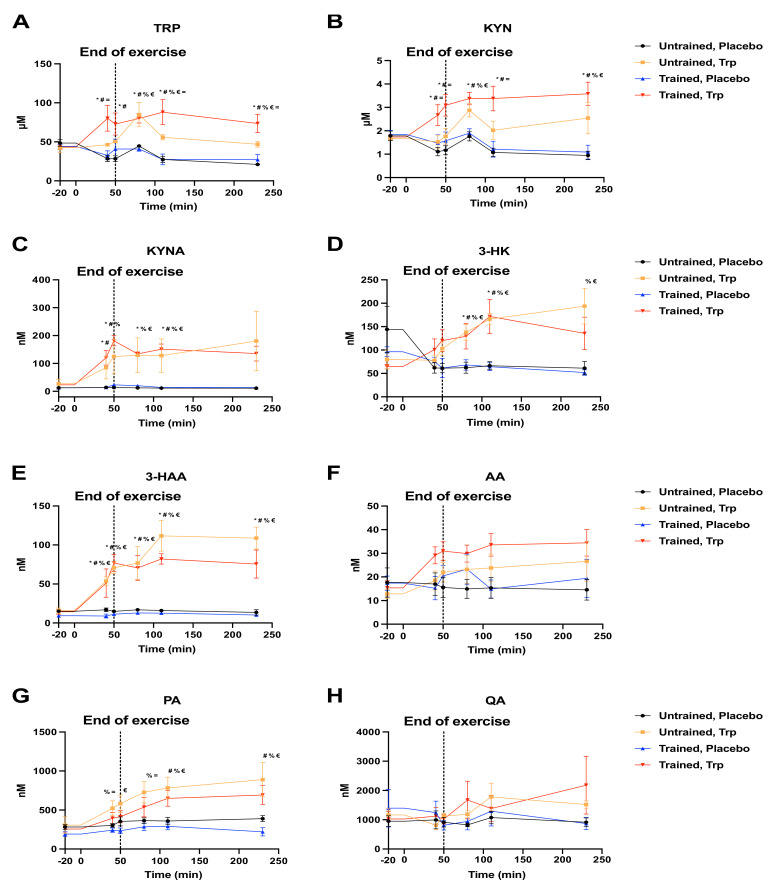
Tryptophan supplementation during exercise in high and low VO_2_ max human volunteers. Each subject performed the exercise protocol twice, either with Tryptophan (TRP) or placebo in a double-blind control setting. After resting, they exercised for 40 min at a given rate corresponding to their 60% VO_2_ max. Thereafter, they performed maximal exercise for another 10 min. In total, subjects were given a 1440 mL drink of flavored water (placebo) or containing 15 mg TRP/kg body weight, 10–15 min before exercise, just before exercise, at 15, 30, and 40 min of exercise, 5, 15, 30, 60, 90, 120, and 150 min of recovery. Blood was collected at rest (20 min before exercise), at 40 min of exercise, at the end of exercise (50 min) and then at 30, 60, and 180 min of recovery. Plasma was used for quantification of the indicated metabolites. (**A**) TRP, Tryptophan. (**B**) KYN, Kynurenine. (**C**) KYNA, Kynurenic acid. (**D**) 3-HK, 3-Hydroxykynurenine. (**E**) 3-HAA, 3-Hydroxyanthranilic acid. (**F**) AA, Anthranilic acid. (**G**) PA, Picolinic acid. (**H**) QA, Quinolinic acid. Data is presented as mean values ± standard error of the mean (SEM). * *p* < 0.05 (untrained placebo vs. trained TRP); # *p* < 0.05 (trained placebo vs. trained TRP); % *p* < 0.05 (untrained placebo vs. untrained TRP); € *p* < 0.05 (untrained TRP vs. trained placebo); = *p* < 0.05 (untrained TRP vs. trained TRP).

Next, we aimed to determine if the exercise-induced changes in circulating KP metabolites we observed in our mouse studies, could also be observed in human volunteers. To that end, we organized a small trial where we recruited volunteers with high or low maximal oxygen uptake (VO_2_ max; Supplementary Table S1) (indicative of muscle oxidative capacity, which positively correlates with KAT expression; [14,15]). The subjects came to the lab in the morning (7 AM) after fasting since 9 PM the previous day. After resting in supine position for 30 min, a catheter was placed in the antecubital vein to enable repeated blood sampling. After resting, they exercised for a total of 50 min; 40 min at a given rate corresponding to approximately 60% of their VO_2_ max followed immediately by maximal exercise for another 10 min. Blood was drawn at rest (20 min before exercise), at 40 min of exercise, at the end of exercise (50 min), and then at 30, 60, and 180 min of recovery. The subjects were given a 120 mL drink 10–15 min before exercise, right before starting exercise, at 15, 30, and 40 min of exercise, 5, 15, 30, 60, 90, 120, and 150 min of recovery. In total, subjects were provided with 1440 mL of either placebo (flavoured water) or an aqueous solution of 15 mg TRP/kg body weight. Plasma was used for KP metabolite quantification as in the mouse study. As expected, the placebo drink did not significantly affect any of the quantified metabolites at any time point and regardless of the subjects’ aerobic fitness level (Figure 6). The TRP drink caused an elevation in circulating TRP and in all analysed KP metabolites, except AA, PA and QA (Figure 6G,H). In trained individuals, TRP and KYN levels increased more than in untrained individuals (Figure 6A,B). No additional differences were observed between the two groups in their response to the TRP supplement.

## 3. Discussion

The search for the most effective exercise and dietary supplementation strategy to improve performance and/or health has yielded numerous studies throughout the years [21]. From the identification that physical exercise and skeletal muscle are important regulators of the KP [14], one important question arises: What are the fate and biological outcomes of TRP supplementation in sedentary vs. exercised individuals? In this study, we aimed to answer this question by using a comprehensive molecular and phenotypic analysis of mice fed a control diet or a TRP-supplemented diet with five-times higher levels of this amino acid. Our results indicate that TRP supplementation was sufficient to increase free wheel running activity over a period of eight weeks, but without affecting in-cage locomotion patterns or leading to more weight loss or molecular evidence of training adaptations, when compared to controls. There are some reports on the effect of TRP supplementation on exercise performance, but they are sometimes difficult to compare due to significant differences in the experimental design. In humans, those reports seem to be somewhat contradictory [22,23]. Recent studies suggest that skeletal muscle serotonin levels are important for exercise performance [24]. Although we did not measure peripheral or central serotonin levels, it is tempting to speculate that the TRP supplementation could have affected serotonin levels, and thus increasing wheel running activity. On the other hand, the number of studies addressing the effects of exercise on TRP catabolism through the KP are constantly increasing [14,15,25,26,27,28,29]. Once again, it is important to consider how the studies were planned when comparing data from the different sources. Timing of sample collection after the exercise or diet intervention is one particularly important aspect, as also highlighted by our data showing how fast the circulating levels of TRP and its catabolites drop as soon as mice went into the light/inactivity phase. This relatively short circulation half-life has also been demonstrated in other species and situations [18,20]. In line with some of those studies, we also observed that exercise led to a decrease in KP neurotoxic metabolites in the TRP-supplementation group. However, that decrease was not reflected in any of the analyzed metabolism or behavior parameters. Together, these results indicate that moderate chronic TRP supplementation does not affect systemic energy metabolism or behavior in mice. It remains to be established if a higher level of TRP supplementation or more strenuous exercise would yield different results.

In the present study we also performed a small trial with human volunteers. For ethical reasons, due to the lack of available data on the effects of long term TRP supplementation in humans (both in terms of metabolism and behavior), the human study design was different from the mouse experiments. We recruited trained and untrained volunteers (with VO_2_ max indicative of skeletal muscle oxidative capacity, which also correlates with KAT levels; [14,15,16]). In this way, we aimed to evaluate what is the fate of a bolus of TRP supplementation during and after endurance exercise, in well-trained vs. untrained subjects, in terms of KP metabolites. The results we obtained show that the TRP supplement resulted in higher TRP and KYN levels than in untrained volunteers. In addition, despite a trend for higher 3-HAA levels in untrained individuals, we did not observe other statistically significant differences between these groups. It is important to note that this was a very small study that should be repeated with a larger cohort. For example, we did not observe the documented increase in KYNA with exercise, which might have been due to the small cohort size or to a specific characteristic of the exercise protocol (e.g., intensity or duration).

Together, our data indicate that moderate TRP supplementation is sufficient to elevate the overall levels of KP metabolites in circulation. Considering the multiple reports on the increase of KYNA following exercise, it would be interesting to further these studies by looking at long-term TRP supplementation in humans during training (which would better mimic the mouse study design). Finally, this study might yield additional information if performed with individuals with dysregulated TRP metabolism.

## 4. Materials and Methods

### 4.1. Animals

Male C57bl/6J mice, 6 weeks old, were purchased from Janvier (Le Genest-Saint-Isle, France) and allowed to adjust to the new animal facility environment for at least a week. Mice were housed in cages maintained at 23–24 °C with 55–65% humidity and a 12 h light–dark cycle. Mice were fed rodent chow R34 (Lantmännen Lantbruk) until the experiment started and then changed to purified diets (i.e., diets formulated with defined amounts of each nutrient, including amino acids; Supplementary Figure S2).

### 4.2. Diets

Purified Diets were purchased from Research Diets Inc. All diets were isocaloric and controlled for specific amino acid profile. The control diet contained 0.2% Tryptophan, as calculated to be the approximate tryptophan content of rodent chow R34 (Lantmännen Lantbruk) and “enriched diet” contained 1% Tryptophan (Supplementary Figure S2).

### 4.3. Free Wheel Running

At 7 weeks of age, mice were weighed daily to get used to handling. At 8 weeks of age, mice were divided into the following groups: single-housed sedentary with control purified diet (SC, 0.2% Tryptophan), single-housed sedentary with purified diet enriched in Tryptophan (ST, 1%), single-housed with access to a running wheel (24/7) with control purified diet (EC), single-housed with access to a wheel (24/7) with purified diet enriched in Tryptophan (ET). After 8 weeks of free wheel running, mice were anesthetized with isoflurane and subjected to dual-energy X-ray absorptiometry (DEXA) scanning. Blood was collected in EDTA coated tubes (Sarstedt) and plasma separated by centrifugation at 2000× *g*, 10 min at 4 °C. Tissues were collected and snap frozen in liquid nitrogen. All mice were sacrificed within the first 2 h of the animal facility’s light cycle.

### 4.4. Behavior

All behavior tests were performed in the light cycle between 8 AM and 3 PM. Mice were moved into the behavior test room and acclimated to the new environment for at least one hour before the start of the first trial. In both tests, mice were recorded using an infrared digital camera and tracked using Ethovision XT software (Noldus, Wageningen, The Netherlands). Discrepancies in the digital analysis were manually checked. The time between behavior tests was never less than 7 days. After 5 weeks of diet, the Open Field test was performed to assess anxiety-like behavior. Briefly, the mouse was placed at the corner of a 40 × 40 cm arena and recorded for 10 min. The arena was wiped with 70% ethanol and left to air dry between each mouse trial.

The Elevated Plus maze test was performed to access for anxiety-like behavior by calculating time spent in open arms. Briefly, the mouse was placed at the center of the maze facing an open arm and recorded for 5 min. The arena was wiped with 70% ethanol and left to air dry between each trial.

### 4.5. Gene Expression Analysis

Total RNA was isolated using Isol-RNA Lysis Reagent (5 PRIME), according to manufacturer’s instructions. Then 1 μg of RNA was treated with Amplification Grade DNase I (Life Technologies, Carlsbad, CA, USA) and from that, 500 ng were used for cDNA preparation using the Applied Biosystem Reverse Transcription Kit (Life Technologies). Quantitative Real-Time PCR was performed in a ViiA 7 Real-Time PCR system thermal cycler with SYBR Green PCR Master Mix (both Applied Biosystems, Waltham, MA, USA). Analysis of gene expression was performed using the ΔΔCt method and relative gene expression was normalized to hypoxanthine phosphoribosyltransferase (HPRT) mRNA levels.

### 4.6. Metabolic Cages

Mice were fed in their home cages for 5 weeks with either control diet (0.2% Tryptophan) or TRP-enriched diet (1% Tryptophan). At week 6 of diet, mice were individually housed in the TSE PhenoMaster home cage system (TSE Systems; Bad Homburg Germany) for 5 days in a controlled environment (12 h light/12 h dark, 24–25 °C, 45% humidity) with ad libitum access to food and water except for a 12 h fasting period during the last dark cycle. Oxygen consumption and carbon dioxide production rate measurements as well as food and water intake were recorded every 3 min. Activity was assessed by beam breaks in TSE Phenomaster systems. Consecutive beam breaks were considered as “ambulatory” and single beam breaks as “non-ambulatory” movements (rearing, grooming etc.). Data were averaged per hour. Measurements from the first 12 h were excluded from analysis as they are considered acclimation period. Data analysis was performed using CalR (www.calrapp.org accessed on 15 June 2021) [30].

### 4.7. Body Composition

Body composition was measured by Dual-Energy X-ray Absorptiometry (DEXA) in a Lunar PIXImus Densitometer (GE Medical Systems, Chicago, IL, USA).

### 4.8. Glucose Tolerance Test

After a 5-h fasting period, we gave each animal an intraperitoneal bolus of glucose (2 g/kg) on sterilized water. We monitored glycaemia at 0, 15, 30, 45, 60, and 120 min after bolus glucose administration. Blood samples were obtained by tail bleeding and measurements were done using a OneTouch Accu-Check glucometer.

### 4.9. Human Exercise Study

Three to five weeks before the study, five untrained (4 males and 1 female) and four endurance-trained subjects (2 males and 2 females) were tested for maximal oxygen uptake. Population characteristics in Supplementary Table S1. Oxygen uptake at three submaximal work rates as well as maximal oxygen uptake (VO_2_max) was determined utilizing an on-line system (Oxycon Pro, Erich Jaeger GmbH, Hoechberg, Germany). The subjects exercised on a mechanically braked cycle ergometer (Monark 839E, Vansbro, Sweden), during the maxtest the work rate was gradually increased until volitional exhaustion (Åstrand PO and Rodahl K, Textbook of Work Physiology, McGraw Hill, New York, NY, USA, 1986). From these measurements, a work rate corresponding to 60% of VO_2_max was calculated for each individual and this work rate was then used in the two supplementary experiments. During the supplementary experiments, the oxygen uptake was measured after approximately 20 min exercise and heart rate monitored continuously (Polar Electro OY, Kempele, Finland).

The average VO_2_max for the untrained group was 40 mL/min/kg body weight and 54 mL/min/kg body weight for the trained group. Subjects were initially asked to perform a familiarization session approximately a week prior to the initiation of the experimental trial. Subsequently, during the experimental trial subjects performed the exercise protocol either with Tryptophan or placebo in a double-blind control setting. The order of the supplement (TRP or placebo) was randomly chosen in a cross-over design and the sessions were separated by 1–3 weeks. The subjects reported to the laboratory at 7 AM after fasting since 9PM the day before. After resting in supine position for 30 min, a catheter was placed in the antecubital vein to enable repeated blood sampling. After resting, the subjects cycled for 40 min at a given rate corresponding to their 60% VO_2_ max. Thereafter, they performed maximal exercise for another 10 min. Blood was drawn at rest (20 min before exercise), at the end of exercise (50 min) and then at 30, 60, and 180 min of recovery. Subjects were given a 1440 mL drink of flavored water (placebo) or containing a total of 15 mg Tryptophan/kg body weight, in 120 mL boluses at 10–15 min before exercise, just before exercise, at 15, 30, and 40 min of exercise, 5, 15, 30, 60, 90, 120, and 150 min of recovery.

### 4.10. Measurement of Metabolites in Plasma

Quantification of KP metabolites were carried out as previously described [31]. Briefly, 150 µL of sample was deproteinized with 10% (*w/v*) trichloroacetic acid in an equal volume. After, deproteinized samples were centrifuged at 4 °C for 10 min at 12,000× g. Next, the supernatant was filtered with 0.22 μm syringe filters (Millex, Merck) for quantification of KP metabolites.

Simultaneous quantification of TRP, KYN, 3-HK, 3-HAA, and AA was carried out using an Agilent ultra-high performance liquid chromatographer (uHPLC). Separation of metabolites was carried out under the stable temperature of 40 °C for 12 min, using 0.1 mM sodium acetate (pH 4.6) mobile phase, with an isocratic flow rate of 0.75 mL/min in an Eclipse Plus C18 reverse-phase column (2.1 mm × 150 mm, 1.8 μm particle size; Agilent Technologies Inc., Santa Clara, CA, USA). TRP, 3-HAA and AA were detected using fluorescence intensity set at Ex/Em wavelength of 280/438 for TRP and 320/438 for 3-HAA and AA. 3-HK and KYN were detected using UV detector set at wavelength of 365 nM.

Quantification of KYNA was performed using a reverse phase C18 column (ZORBAX XDB, 4.6 × 100 mm; Agilent Technologies Inc., Santa Clara, CA, USA) in an Agilent high performance liquid chromatographer (HPLC) system. The mobile phase (50 mM sodium acetate, with 50 mM zinc and 5% *v/v* HPLC grade acetonitrile, pH 5.2) was run at an isocratic flow rate of 1 mL/min with a stable temperature of 35 °C for 8 min. KYNA was detected using fluorescence intensity set at Ex/Em wavelength of 344/388. Agilent OpenLAB CDS Chemstation (Edition C. 01.04) was used to analyse all above the chromatograms. The inter- and intra-assay coefficient of variation is within the acceptable range of 3–7%.

PA and QA was quantified using the Agilent 7890 A Gas Chromatography (GC) coupled with Agilent 5975 C mass spectrometry detector and Agilent 7693 A autosampler (Agilent Technologies Inc., Santa Clara, CA). The separation of PA and QA were performed with a DB-5MS column, 0.25 μM film thickness, 0.25 mm × 30 m capillary column (Agilent Technologies Inc., Santa Clara, CA) for 12 min. A series of deuterated and non-deuterated standards for PA and QA were used for a six-point calibration curve to interpolate the quantity of the sample readout using Agilent GC/MSD ChemStation software (Edition 02.02.1431).

Additional metabolite quantifications were performed at Bevital AS (Bergen, Norway), and a detailed description of the analytical method used has been previously published [32].

To determine the best time to end experiments relative to the beginning of the light cycle in the animal facility, mice were kept in control or TRP-enriched diet for 2 weeks. At the end of the second week, mice were sacrificed, and blood collected at 6 AM (start of light phase) or 10 AM.

### 4.11. Statistical Analysis

All data are given as mean +/− SEM unless specified. Differences between 2 groups were assessed by unpaired student *t*-test. Three or more group analysis were evaluated by one-way ANOVA, followed by multiple comparisons test with Tukey correction. Any outliers were identified using the ROUT method [33].

For the human data, group comparison was performed using one-way ANOVA followed by Fisher’s LSD post hoc test was performed. *p* < 0.05 was considered statistically significant for all analysis. Statistical analysis was performed using GraphPad Prism v9.

## Data Availability

All data is contained within the article or supplementary materials.

## Supplementary Materials

The following are available online at https://www.mdpi.com/article/10.3390/metabo11080508/s1, Figure S1: Schematic representation of the kynurenine pathway, Figure S2: Composition of the different diets, Figure S3: Metabolic analysis of expenditure changes in animals fed with control diet (SC, 0.2% TRP) or TRP enriched diet (ST, 1% TRP) diets, Figure S4: Kynurenine pathway metabolite analysis to determine the optimal time to terminate the experiment, Figure S5: Moderate TRP-supplementation does not influence open field behavior test results, Figure S6: Moderate TRP-supplementation does not influence elevated plus maze behavior test results, Table S1: Human volunteer population characteristics. Weight (kg). BMI, Body mass index. Age (yr). HR, Heart rate. TRP, tryptophan. RER, Respiratory exchange ratio.
