# Supplementary material for: Effects of Tryptophan Supplementation and Exercise on the Fate of Kynurenine Metabolites in Mice and Humans
Source: Metabolites. 2021 Aug 3;11(8):508. doi: 10.3390/metabo11080508 (PMC8400416; doi:10.3390/metabo11080508)
Supplement: Supplementary file 1 [file metabolites-11-00508-s001.zip › metabolites-1317272-supplementary.pdf]

Figure S1

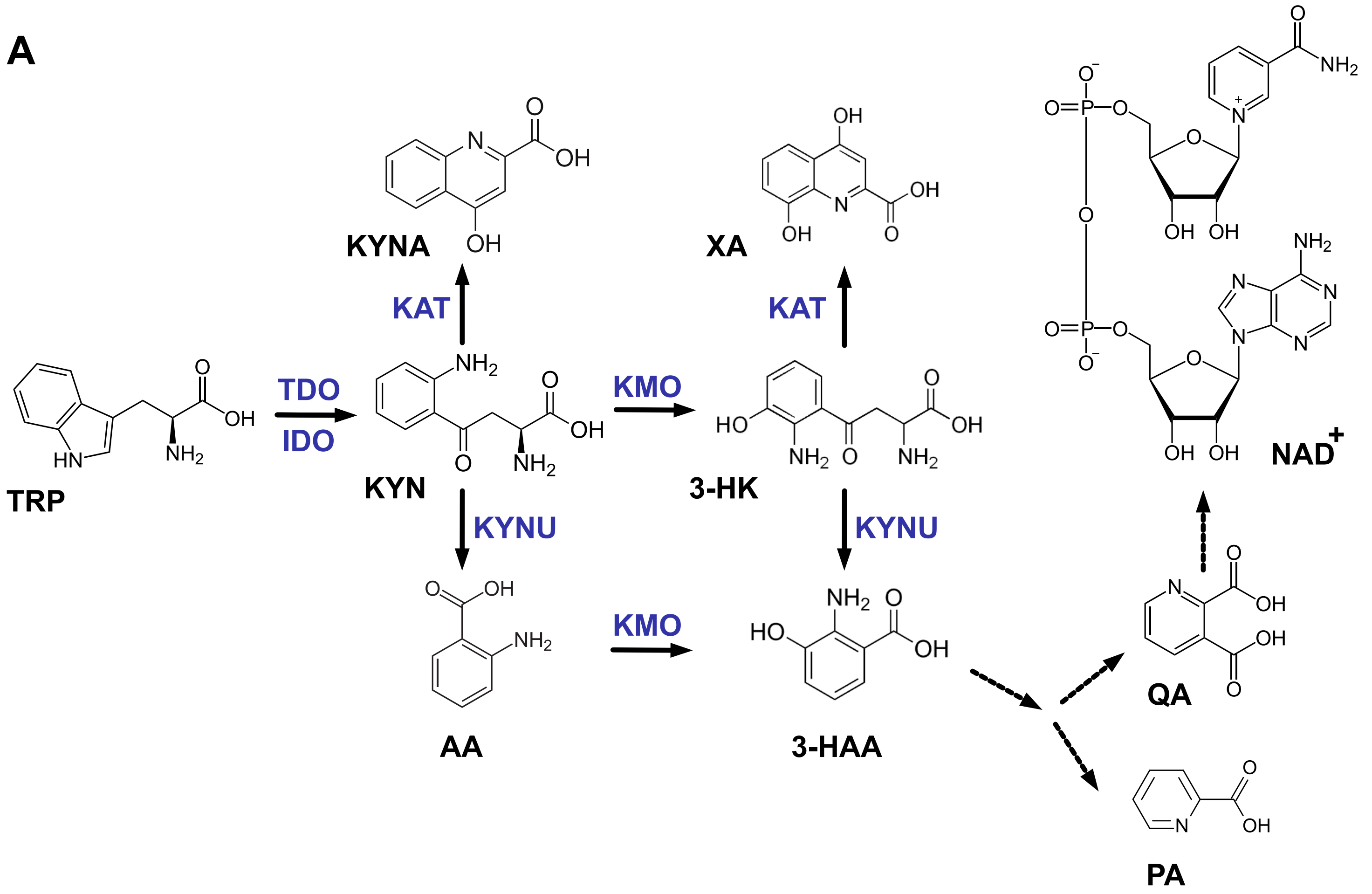
A
TRP
TDO
IDO
KYN
KAT
KYNA
KMO
3-HK
XA
KYNU
AA
3-HAA
QA
PA
NAD+

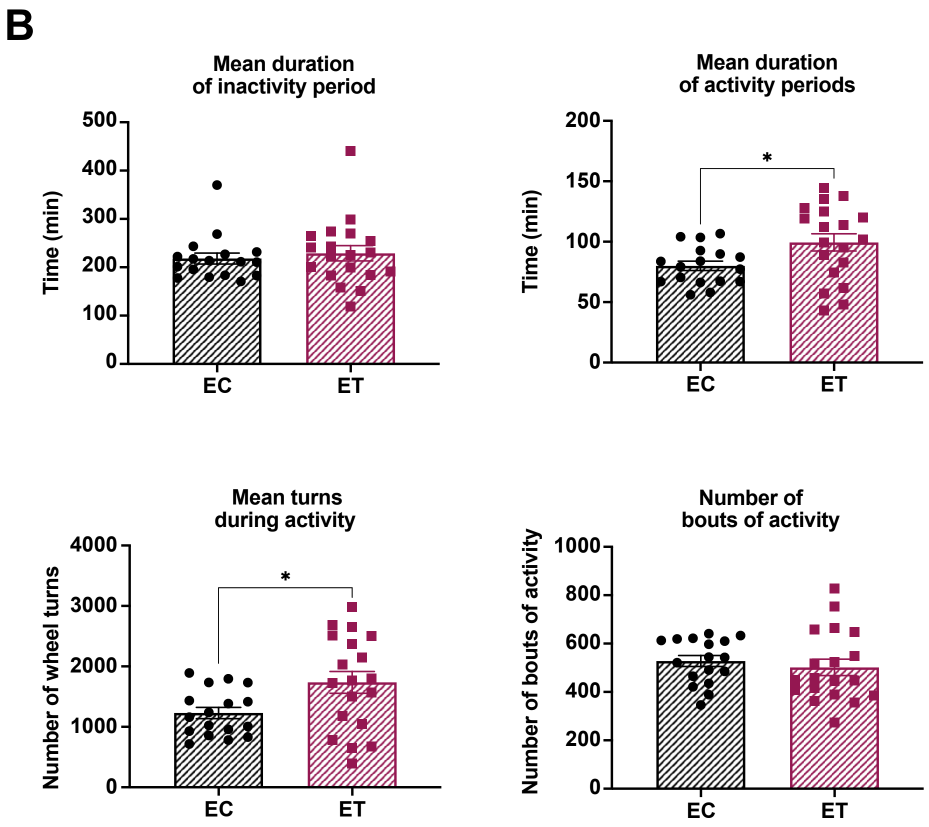
B
Mean duration of inactivity period
Time (min)
Mean duration of activity periods
Mean turns during activity
Number of wheel turns
Number of bouts of activity
Number of bouts of activity
EC
ET
*

Figure S2

L-Amino acid rodent diet with different levels of Tryptophan

Product #	A10021B		A16042602	
	g%	Kcal %	g%	Kcal %
**Protein**	17	18	18	18
**Carbohydrate**	69	71	68	70
**Fat**	5	12	5	12
Total		101		100
kcal/g		3.9		3.9
**Ingredient**	**g**	**kcal**	**g**	**kcal**
L–Arginine	10	40	10	40
L–Histidine–HCl–H_20	6	24	6	24
L–Isoleucine	8	32	8	32
L–Leucine	12	48	12	48
L–Lysine–HCl	14	56	14	56
L–Methionine	6	24	6	24
L–Phenylalanine	8	32	8	32
L–Threonine	8	32	8	32
**L–Tryptophan**	**2**	**8**	**10**	**40**
L–Valine	8	32	8	32
L–Alanine	10	40	10	40
L–Asparagine–H_2O	5	20	5	20
L–Aspartate	10	40	10	40
L–Cystine	4	16	4	16
L–Glutamic acid	30	120	30	120
L–Glutamine	5	20	5	20
Glycine	10	40	10	40
L–Proline	5	20	5	20
L–Serine	5	20	5	20
L–Tyrosine	4	16	4	16
L–Aminoacids, Total	170	680	178	712
Corn starch	550.5	2202	542.5	2170
Maltodextrin 10	125	500	125	500
Sucrose	0	0	0	0
Cellulose	50	0	50	0
Corn Oil	50	450	50	450
Mineral Mix S10001	35	0	35	0
Sodium bicarbonate	7.5	0	7.5	0
Vitamin Mix V10001	10	40	10	40
Choline bitartarate	2	0	2	0
Blue Dye, FD&C #1	0.05	0	0.025	0
Red Dye, FD&C #40	0.00	0	0.025	0

Figure S3

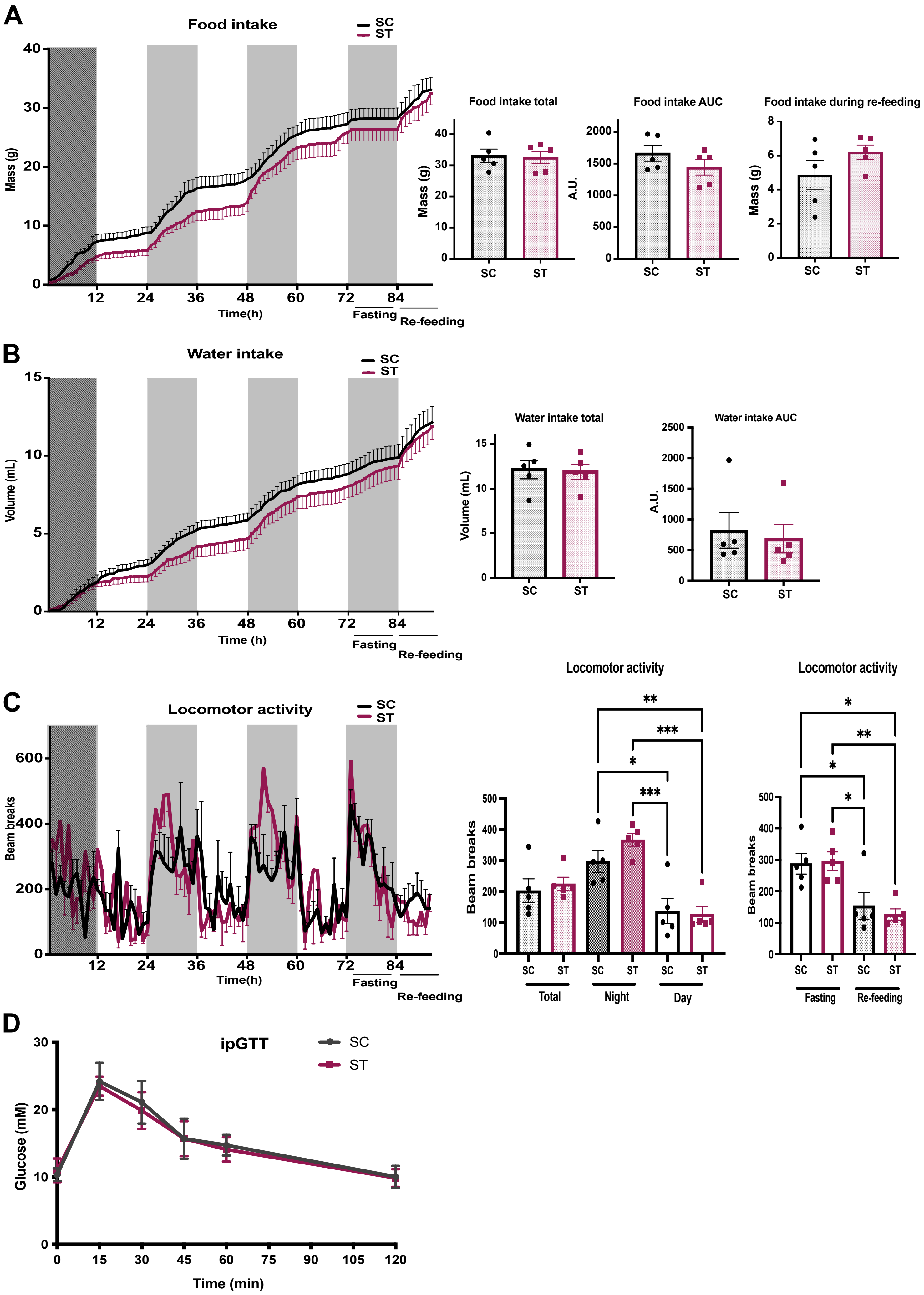
A
Food intake
SC
ST
Mass (g)
Time(h)
Fasting
Re-feeding
Food intake total
Food intake AUC
Food intake during re-feeding
A.U.
B
Water intake
Volume (mL)
Time (h)
Water intake total
Water intake AUC
C
Locomotor activity
Beam breaks
Total
Night
Day
D
ipGTT
Glucose (mM)
Time (min)

Figure S4

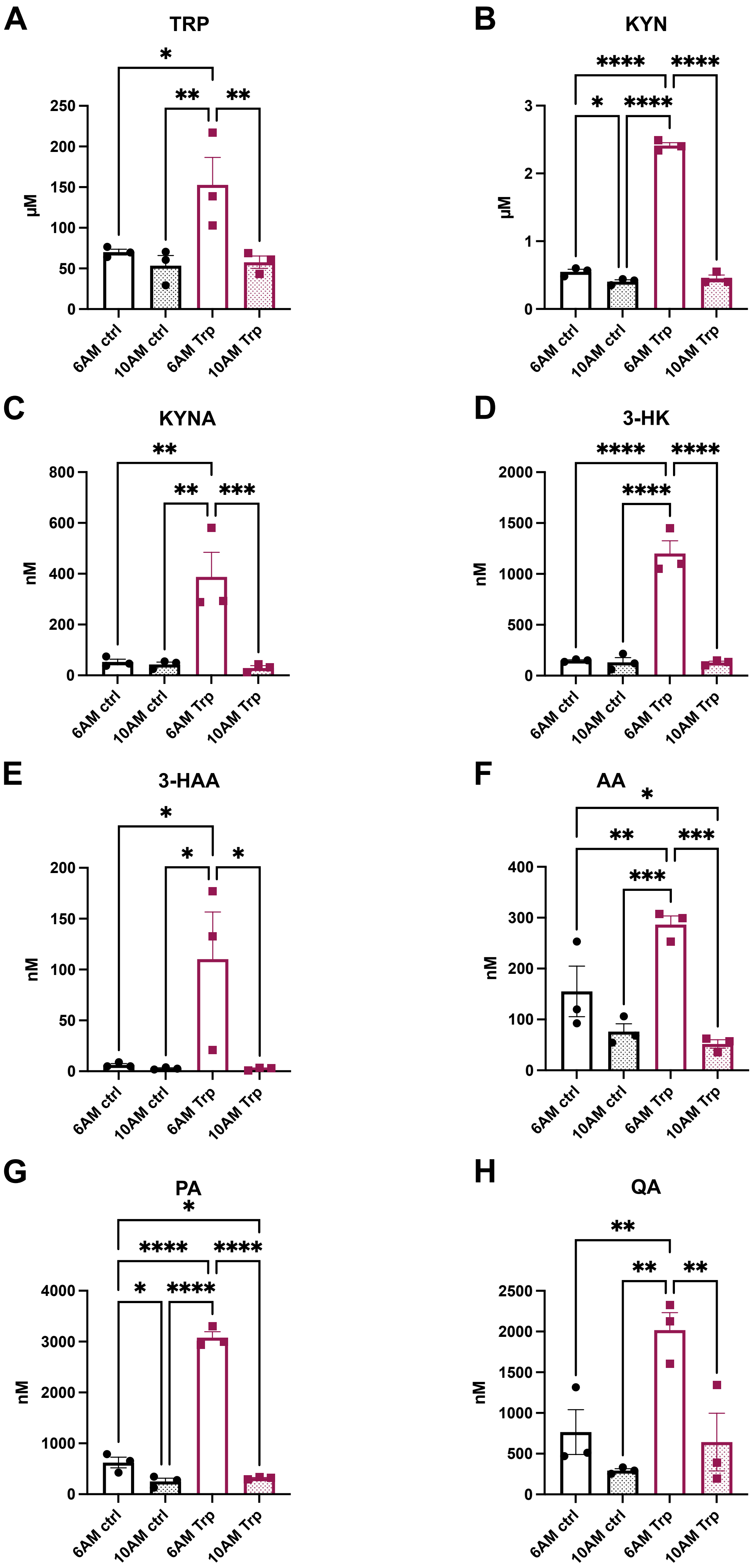

A
TRP
μM
B
KYN
μM
C
KYNA
nM
D
3-HK
nM
E
3-HAA
nM
F
AA
nM
G
PA
nM
H
QA
nM
6AM ctrl
10AM ctrl
6AM Trp
10AM Trp

Figure S5

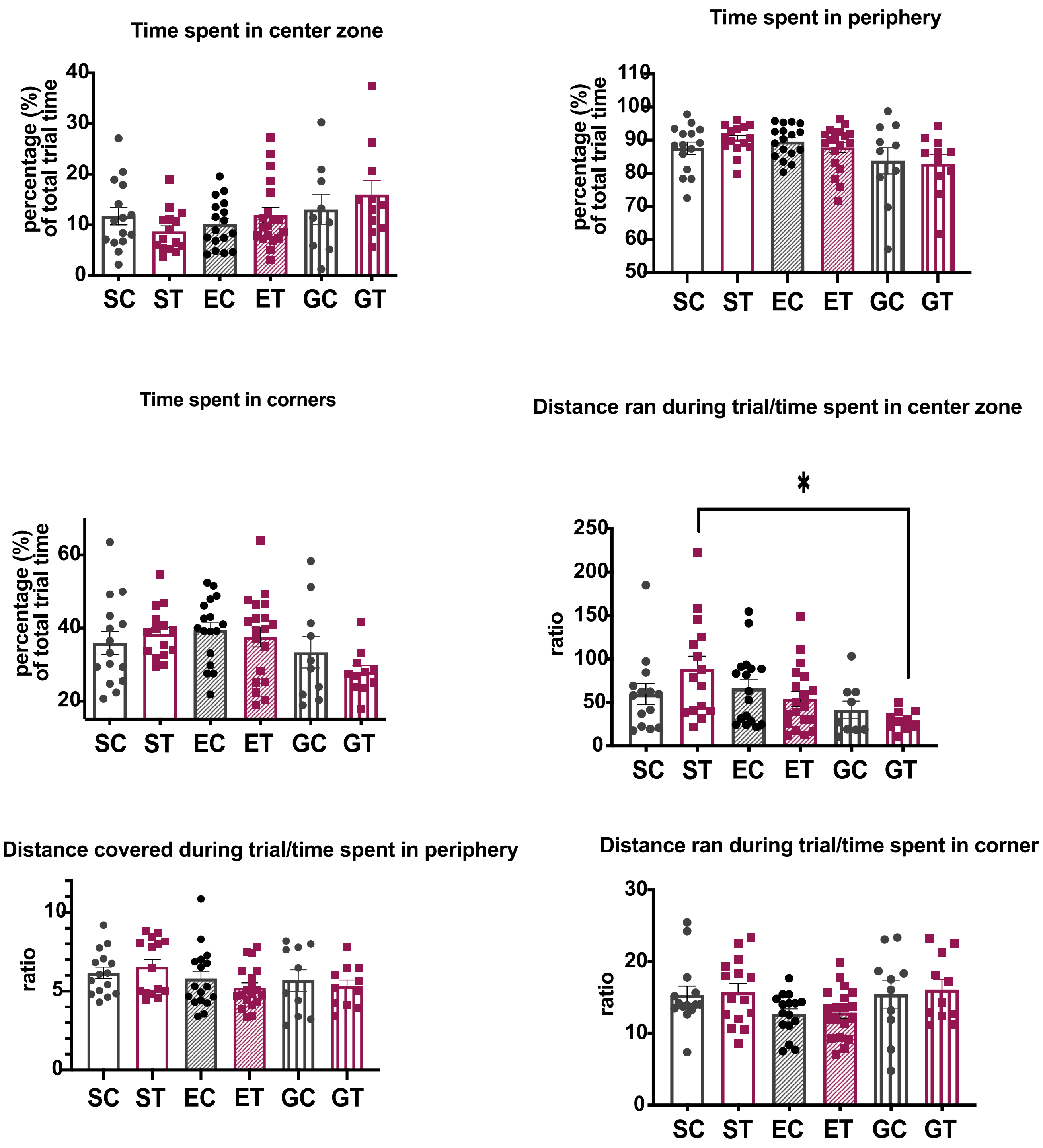

Time spent in center zone
percentage (%) of total trial time
Time spent in periphery
percentage (%) of total trial time
Time spent in corners
percentage (%) of total trial time
Distance ran during trial/time spent in center zone
ratio
*
Distance covered during trial/time spent in periphery
ratio
Distance ran during trial/time spent in corner
ratio
SC ST EC ET GC GT

Figure S6

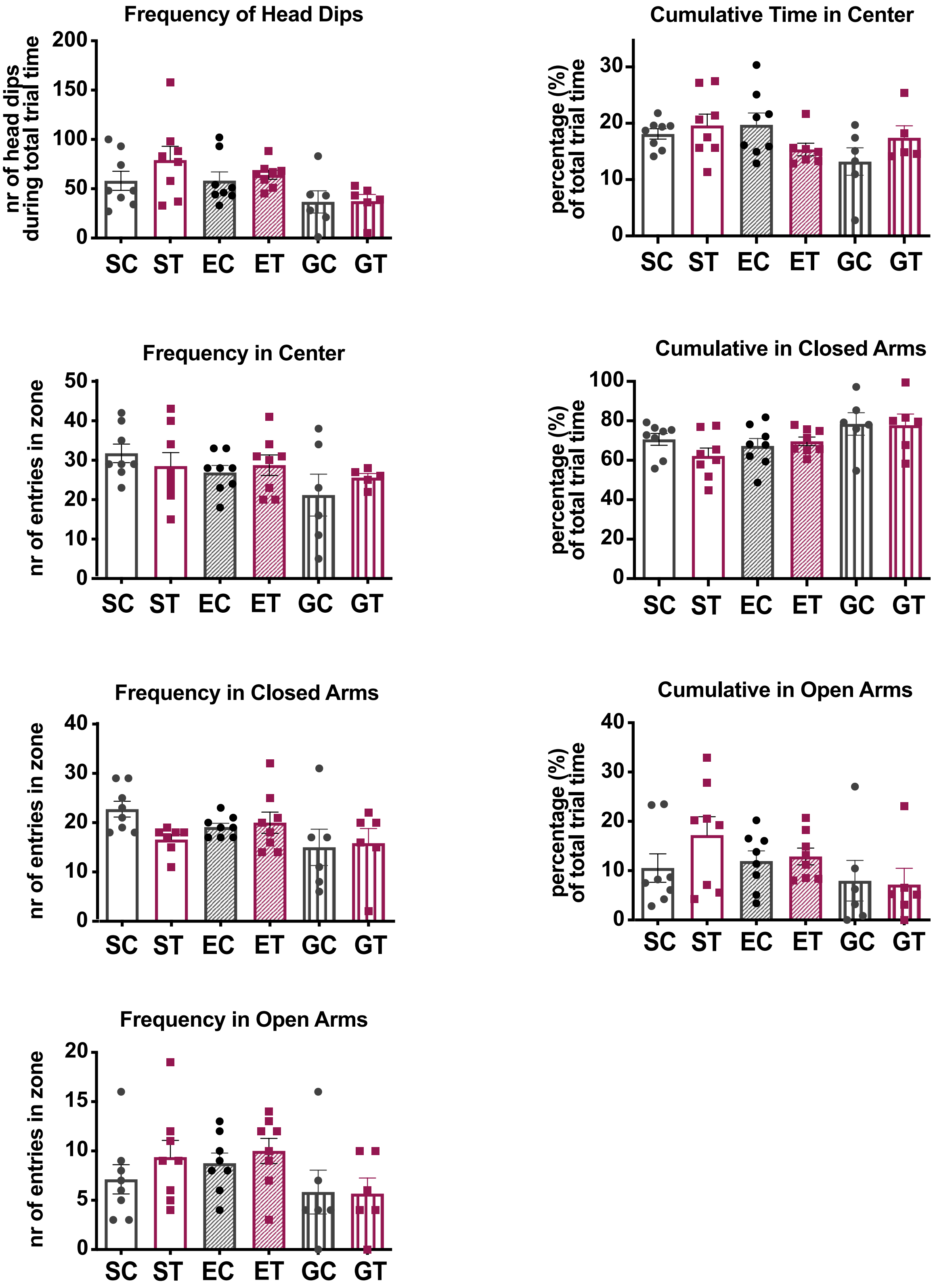
Frequency of Head Dips
nr of head dips during total trial time
200
150
100
50
0
SC ST EC ET GC GT
Cumulative Time in Center
percentage (%) of total trial time
30
20
10
0
SC ST EC ET GC GT
Frequency in Center
nr of entries in zone
50
40
30
20
10
0
SC ST EC ET GC GT
Cumulative in Closed Arms
percentage (%) of total trial time
100
80
60
40
20
0
SC ST EC ET GC GT
Frequency in Closed Arms
nr of entries in zone
40
30
20
10
0
SC ST EC ET GC GT
Cumulative in Open Arms
percentage (%) of total trial time
40
30
20
10
0
SC ST EC ET GC GT
Frequency in Open Arms
nr of entries in zone
20
15
10
5
0
SC ST EC ET GC GT

Supplemental Table S1

Training status of volunteers		Untrained	Trained
Weight		76.3 +/-20.3	64.2 +/-2.9
BMI		23.6 +/-5.8	22.3 +/-2.6
Age		31.4 +/-4.7	24.8 +/-6.3
Average VO2 max	(l/min)	3 +/-0.6	3.495 +/-0.7
	(ml/min. kg)	39.9 +/-4.2	54.2 +/-8.9
% of VO2 max	Placebo	60.4 +/-1.9	59 +/-3.6
	TRP	60.5 +/-1.5	59.4 +/-2.3
max HR		188.6 +/-3.4	194 +/-13
RER	Placebo	0.9 +/-0.06	0.9 +/-0.02
	TRP	0.9 +/-0.06	0.92 +/-0.02
HR (0-40min)	Placebo	132.4 +/-6.2	145 +/-13
	TRP	133.1 +/-9	147.3 +/-11
Work (km) 40-50min	Placebo	6.6 +/-0.4	7.2 +/-0.3
	TRP	6.5 +/-0.5	7.2 +/-0.2

Values are presented as mean +/- SD
